# Translational Difficulties in Querying Rats on “Orientation”

**DOI:** 10.1155/2019/6149023

**Published:** 2019-12-30

**Authors:** Aliz Judit Ernyey, Eszter Bögi, Ferenc Kassai, Imola Plangár, István Gyertyán

**Affiliations:** ^1^MTA-SE NAP B Cognitive Translational Behavioural Pharmacology Group, Department of Pharmacology and Pharmacotherapy, Faculty of Medicine, Semmelweis University, Budapest 1089, Hungary; ^2^Institute of Cognitive Neuroscience and Psychology, Research Center for Natural Sciences, Hungarian Academy of Sciences, Budapest 1117, Hungary; ^3^Institute of Experimental Pharmacology and Toxicology, Centre of Experimental Medicine, Slovak Academy of Sciences, Bratislava 841 04, Slovakia

## Abstract

The aim of this study was to translate the “orientation” query of the ADAS-Cog inventory to rats and to investigate whether they can determine which time of the day they are. For this purpose, we established a modified Morris water-maze navigation task where the escape platform was placed onto various locations at different times of the day: “morning”, “noon” and “evening”. In each of these sessions rats swam a “query” trial and a “confirmatory” trial, 30 min apart. Lister Hooded rats randomly chose among the three possible target locations, while Long Evans rats partly followed a win-stay strategy by preferring to visit first to the platform position of the previous session. Despite simplifying the task to a morning–evening discrimination, Lister Hooded rats continued searching by chance, while Long Evans rats switched to the mentally less demanding random strategy. We then inserted a board into the pool which required longer swimming path from the animals when they were correcting an initial wrong choice, but this modification did not result in a change in the above strategies. Lastly, in a separate group of Long–Evans rats, the training conditions were modified inasmuch an incorrect choice was definitely punished by impeding the animals to correct it and confining them to a platform-free part of the maze for the whole trial period. However, even these stricter conditions were not sufficient to make the rats distinguish times of the day. The observed lack of time discrimination may source from an evolutionary built in mechanism characteristic for the rat species or this ability may have only been lost in laboratory rats.

## 1. Introduction

Patients suffering from Alzheimer's disease (AD) have difficulties in remembering recent events that took place at a particular time and place. This indicates one of the first cognitive and behavioral symptoms which is the deterioration of episodic memory. Tulving conceptualized episodic memory as a conscious awareness of a unique prior experience, and not only as the knowledge about what a particular event was and where and when it occurred [1]. As such, episodic memory is conceived to be unique to humans; nevertheless, many investigators could reveal an episodic-like memory system in animals akin to that of humans with respect to fulfilling the criteria of the what-where-when triad. Clayton and Dickinson provided conclusive behavioral evidence of a so-called “episodic-like” memory in scrub jays that remembered in a food-caching paradigm when, where and what kind of food items were stored [2].

Several attempts were made to set up models of episodic memory in laboratory animals. Dere et al. designed a three-trial object exploration task in mice, in which the investigation of object recognition memory, location memory, and temporal order memory was combined. With varying the entity and location of objects it was shown that mice could reflect memory of “what”, “when,” and “where”, that fulfills behavioral criteria of an episodic-like memory [3]. The same group demonstrated that this novelty-preference paradigm also works with rats [4].

Babb and Crystal also demonstrated the functioning of an episodic-like memory in rats in a radial-arm-maze paradigm. In their task “where” meant various locations (arms) where food can be found, “when” was set by different timings of the acquisition and test phases of the task and “what” was represented by the different quality of food at the different locations [5]. Babb and Crystal showed that in this type of task rats relied rather on an interval-timing than a circadian time-of-day strategy [6]. Roberts et al. supported this conclusion by demonstrating that rats were able to remember “how long ago” a specific event happened but could not specify “when” it occurred [7]. On the other hand, Zhou and Crystal came to the conclusion that rats could equally well use the circadian time of day strategy in a similar task. The latter finding suggests that rats not only can distinguish relative time periods but can also tell absolute time, i.e. a specific time of the day [8].

Although episodic memory remarkably deteriorates in AD [9], the most commonly used tests for measuring human cognitive performance in the patients like ADAS-Cog [10], (http://memoryworks.org/screening/ADAS/ADAS_Packet.pdf), MMSE [11], (http://www.dementiatoday.com/wp-content/uploads/2012/06/MiniMentalStateExamination.pdf) or MoCA [12], (https://www.mocatest.org) do not include a specific item for episodic memory. The so-called “orientation” task, in which subjects have to tell their names, the actual month, date, year, day, time of day, season, and place stands the closest to episodic memory inasmuch as it reflects a continuously updated knowledge on the when and where component of episodic memory but without relating to any specific event (“what”). As such, intact time-place orientation is a prerequisite to a functional episodic memory system.

Our aim was to translate the “orientation” query of these inventories to rats and to study if rats are able to determine which time of the day they are. For this purpose, we modified the Morris water navigation task in that the escape platform was placed onto different locations depending on the time of the day.

This type of paradigms and similar appetitively motivated assays are known as time-place learning (TPL) in the literature. While there are several examples of successful time-place learning in animals, rats are not especially good in this learning ability [13]. TPL can be considered as a particular case of episodic-like memory in which the “what” component is preset for the animal (finding food or an escape route) and this fixed event (episode) should be connected to a particular place-time (“where”–“when”) pairing. Interestingly, studies on time-place learning, which have come from comparative behavioral research with an evolutionary approach only recently reached an intersection with the studies on episodic-like memory primarily with a translational approach [14, 15].

The current study is part of a bigger project, namely to establish a complex rodent cognitive test battery of high translational value [16]. In this cognitive model system animals take part parallel in many different cognitive tasks across their lifespan [17, 18]. The applied assays aim at modelling the human cognitive domains that characterize the cognitive deficit patterns of psychiatric and neurodegenerative diseases, i.e., Alzheimer's disease. One of these assays should serve as a test for episodic-like remembering.

## 2. Materials and Methods

### 2.1. Subjects

Experiments were conducted with three cohorts of animals: 36 male 3–7 month-old Lister Hooded (Charles River, Italy) rats, 35 male 8-month-old Long Evans (Janvier, France) rats and other 18 male 18 month old Long Evans (“LEJ2”) rats. All of them previously completed the standard test of Morris water navigation task. They participated in a certain version of episodic memory test at their age of 9–10 months (Lister Hooded, LH), 18 months (Long Evans, LE) or 20 months (other 18 Long Evans, “LEJ2”).

Animals were kept in reversed light-dark cycle (dark phase from 4:00 am until 4:00 pm) and were fed with commercial pellet rat feed R/M-Z+H produced by SSniff Spezialdiäten GmbH. They had access to limited amount of food (45 g/day/3 cage-mates) supplied at the end of the dark phase, at 3:30 pm. Water was available ad libitum. The weight of the rats were in the range of 250–319 g and 371–544 g in case of Lister Hooded rats, 325–440 g and 377–556 g in case of Long Evans rats, while “LEJ2” rats weighed 415–518 g and 413–505 g at the beginning and at the end of the test, respectively. They were housed in groups of three rats in 1500 cm^2^ polycarbonate cages, in enriched environment with paper tube and wooden bricks to chew and were habituated to handling intensively throughout the measurements. The animals were studied lifelong in several food-rewarded cognitive tests. As dietary restriction is known to enhance cognitive performance, health and physical condition [19], we applied a limited food access regime in their feeding. This regime also allowed the rats to maintain their motivation to get rewards during the entire active period.

The experiments were authorized by the regional animal health authority in Hungary (resolution number PEI/001/3572-4/2014) and conformed to the Hungarian welfare law and the EU 63/2010 Directive.

### 2.2. Apparatus and Training Procedure

A black circular pool (diameter 190 cm, depth 60 cm) was filled up with about 37–39 cm tap water (23 ± 1°C). In the pool, a black platform (diameter 10 cm, height can be set between 36.5 and 38.5 cm) was placed with its surface 0.5 cm below the water. The platform was located in one of the four ordinal (north-west (NW), north-east (NE), south-west (SW), south-east (SE)) positions, about 40 cm away from the outside edge of the pool. Extra-maze cues (colored shapes on the wall of the lab, furniture, water tap, door, light source, the experimenter himself/herself) were fixed in the lab in order to facilitate the orientation of the animals. The experiment was carried out on about 50 lx luminance intensity. Escape latency and swimming path were recorded using Smart v3.0 video tracking system software (Panlab, Spain).

Fecal boli were removed from the water after each trial. Water was exchanged and the pool was thoroughly cleaned after every week of testing.

### 2.3. Experimental Procedures

#### 2.3.1. Standard Morris Experiment

Rats were placed into the water facing the rim of the pool and were given 180 s to escape to the hidden target. Once the animal was released at the starting point, the experimenter always returned to the same position and he/she stayed there until the trial was completed. If the animal did not reach the platform within 180 s, it was guided there with gentle hand movements by the experimenter. It was allowed to remain on the platform for 30 s, afterwards was dried by a cloth and returned to its cage.

First, the standard test was performed, in which all of the animals were trained for four days to escape onto the hidden platform from each of the cardinal starting positions (north, west, south, or east) in the maze. The rats completed 3 daily trials between 10:00 am and 1:00 pm with an intertrial interval of 30  min. The platform was placed in the south-east quadrant of the pool.

#### 2.3.2. 3-Platform Experiment

This experiment was performed with 12 Long Evans rats and 12 Lister Hooded rats in a modified version of the standard test. Rats were swimming in three sessions a day: at 5:00 am (“morning” for rats), 10:00 am (“noon”) and 3:00 pm (“evening”). The platform was located at north-east in the maze every morning, north-west at noon and south-west in the evening. Each session consisted of two trials. The first one was the “query trial” (trial 1); performance of the animals in this trial formed the outcome measures. The second trial, commencing 30 min after the first one, served as a “confirmatory” trial (trial 2) regarding the daytime position of the platform. Animals were placed at one of the cardinal starting positions (north, west, south, east) at the beginning of each trial. Lister Hooded rats were run over the course of 2 × 4 consecutive days for 2 weeks starting directly after the weekend of the standard Morris experiment. Long Evans rats were tested for 3 weeks with a break at weekends, starting one week after completing the standard Morris experiment. Escape latency, time of first visits to the other possible “active” target zones and number of entries into the formerly used target zone (in standard experiment) were measured and pathway of swimming was tracked. The primary outcome measure was the rank of visit to the current target zone among the first visits to the possible target locations.

Care was taken in this and in subsequent experiments that the measurements are performed by the same experimenter or when more experimenters carried out the test, they were randomly changing between the different sessions. This way, the person conducting the test could not serve as a cue for the time-place learning test. It was also important not to give additional temporal cues to the rats by testing them in other cognitive tasks between the daily swimming sessions.

#### 2.3.3. 2-Platform Experiment

In order to facilitate the performance of the animals the task was simplified for the same 12 Lister Hooded rats used in the 3-platform-experiment. We reduced the number of daily sessions to a noon session at 9:00 am and an evening session at 3:00 pm with 2 trials (30 min apart) per session. The platform remained in the north-west quadrant at noon and in the south-west quadrant in the evening session, although they were placed a few centimeters farther from the starting point, which was at the “east” part of the maze. So, rats needed to swim a bit longer distance to reach the platform. Both target locations were situated in an equal distance from the starting point. When rats swam directly to the platform without entering the other target zone they were rewarded with food pellets, while they were on the platform. This experiment was started on the week right after the 3-platform experiment was finished. It ran 2 × 4 days long and after one month break another 5 days of training was carried out.

#### 2.3.4. 2-Platform + Board Experiment

To increase the swimming distance between the possible target zones, which were in the north-west quadrant at 6:00 am (“morning”) and in the south-west quadrant at 2:00 pm (“evening”), a separation board (height 60 cm) was placed in the pool along the east-west diameter. Each animal was released at the “east” starting point, where a 20 cm wide corridor between the separation wall and the rim of the pool served as a gateway between the two halves of maze. 12 Lister Hooded rats after taking part in three previous experiments (standard Morris water-maze, 3-platform- and 2-platform experiment) and subsequently having 15 weeks of break, were retrained in this experiment for 3 weeks (5 + 5 + 3 training days). Other 24 Lister Hooded male rats that completed the standard Morris experiment 3 months before, but did not take part in 3-platform- and 2-platform experiments, also performed the test with separation board according to the following schedule: half of these animals swam on 4 consecutive days only in the morning, then 4 days only in the evening, or vice versa for the other half of the group. After these 2 × 4 pretraining days, the training continued in week 3 with alternating morning or evening sessions for the rats. Each rat had one daily session with 2 trials, either in the morning or in the evening. On the first training day, rats were tested at the same time of the day when they had been trained on the last pre-training occasion. Afterwards, morning and evening sessions were alternated daily for 23 days.

Furthermore, 35 Long Evans rats that were not involved in 3-platform- and 2-platform-experiments, were trained for this task for 7 weeks (4 consecutive days per week) but the pre-training period was omitted. This training followed 10 months after their standard Morris experiment. Each animal swam either in the morning or in the evening, always one session per day. The animals swam always 2 trials per session. In case of swimming directly to the target, they were rewarded with three pellets.

#### 2.3.5. 2-Platform + Closed Board Experiment

As a reminder for “LEJ2” animals the standard Morris experiment was repeated in one morning session and one evening session on two respective days. Following that, the animals were tested in the 2-platform + board setup using the separation board in the maze, like in the previous experiment. They performed the task both in the morning and in the evening with two trials/session for five days. From the 6^th^ training day, in case rats chose the wrong direction, i.e. swam to the “evening” platform position (SW) in the morning or to the “morning” platform position (NW) in the evening, the corridor between the two halves of the maze was closed. That is, they could not correct their initial wrong choice by swimming back to the correct location. First choosing the correct direction but turning back to the other half of the maze before finding the platform was considered as incorrect choice, thus the corridor was also closed. The animals were confined in the platform-free half of the maze for 5 min in trial 1 and 3 min in trial 2, before taken out from the maze. This experiment was repeated for 12 days both in the morning and in the evening with 2 trials/session.

### 2.4. Statistical Analysis

Escape latencies and number of entries into previous target zones were analyzed with repeated measures ANOVA followed by Duncan post hoc test. Escape rank to the target was compared to chance level with *χ*^2^ test. Latencies on day 13 in 2-platfom-experiment and 2-platform + board experiment were compared with dependent samples *t*-test.

## 3. Results

### 3.1. Standard Morris Water-Maze Experiment

**Figure 1:**
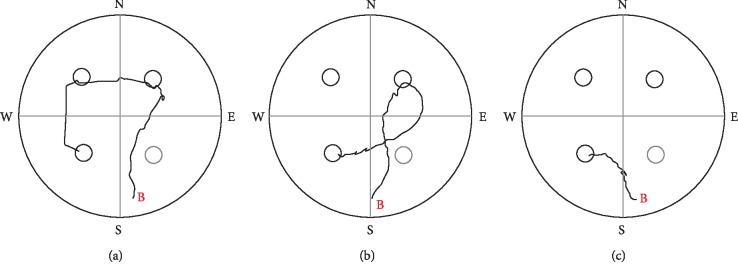
Learning performance in the standard Morris water-maze test. Orange (dark gray) triangle symbols and dashed lines represent the performance of Lister Hooded (LH) rats and black circle symbols and solid lines represent the performance of the Long Evans (LE) group. Mean of individual means of three daily trials ± SEM are plotted. ∗indicates significant difference between LE and LH groups.

All the 36 Lister Hooded and all the 35 Long Evans rats successfully learned the standard Morris test as shown by the significant decreases in the latency to find the hidden platform across 4 days. (*F*_(3, 210)_ = 225.8, *p* < 0.001; Figure 1). The Lister Hooded and Long Evans learning curves run similarly. The “days” × “strain” interaction was significant (*F*_(3, 210)_ = 3.3, *p* < 0.05). Duncan post hoc test revealed that the latencies of LH and LE groups differed significantly only on day 1 (*p* < 0.01) but not on the other days.

### 3.2. 3-Platform Experiment

**Figure 2:**
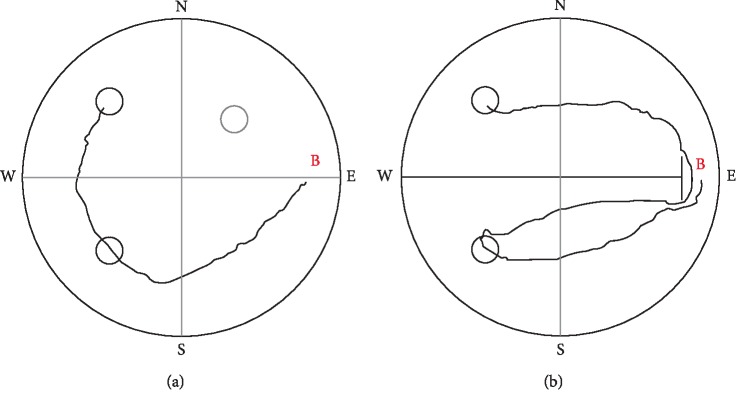
Daily mean ± SEM escape latency values of the first trials in the morning, noon and evening sessions in the 3-platform experiment. Symbols are as in Figure 1.

Out of the animals that were trained in the standard Morris water maze task, 12 LE and 12 LH rats were used for the 3-platform experiment. The daily mean of escape latency to the actual target zones diminished continuously through the experiment period. The performance of the two groups did not differ significantly according to the repeated measures ANOVA performed on data of the first 8 days (“days” effect *F*_(7, 154)_ = 18.5, *p* < 0.001; Figure 2).

**Figure 3:**
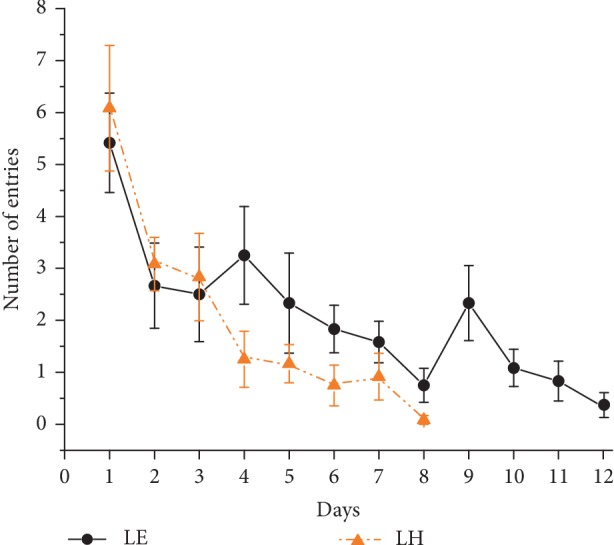
Number of visits ± SEM per day to the former target location (used in the standard setup) in 3-platform experiment in the “query trial”. Symbols are as in Figure 1.

**Figure 4:**
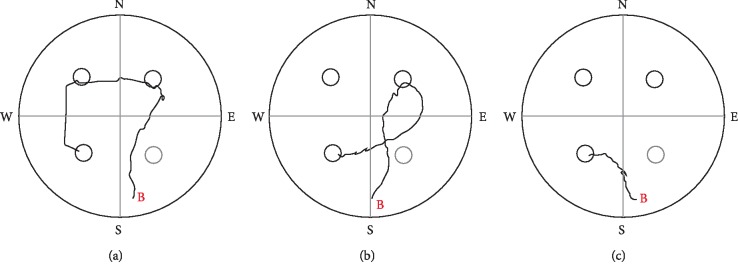
Sample tracks to the “evening” target position from the “South” beginning point (B) of LH rats on day 3. (a) sequentially visiting the three potential target zones (escape latency: about 8  s), (b) swimming through one potential target place before reaching the platform (escape latency: about 6  s), (c) swimming directly to target (escape latency: about 3  s). The dotted circle demonstrates the target zone of the standard Morris experiment, the rats were previously trained for.

During the training the animals gradually ignored the formerly used platform position shown by the significant decrease in the number of entries into the south-east target zone (*F*_(7, 154)_ = 10.9, *p* < 0.001) for both LH and LE rats in the “query trial” (Figure 3). ANOVA was performed on the data of the first 8 days and revealed no significant “strain” effect and interaction. By the end of the training rats adapted the strategy of sequentially visiting the three potential target zones to find the platform (Figure 4).

**Figure 5:**
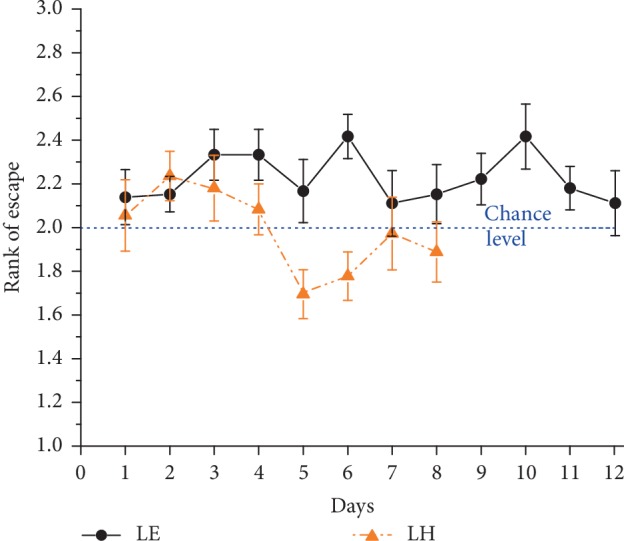
Mean rank ± SEM of visits to the actual target zone out of the three potential locations in trial 1 of the 3-platform experiment. (Swimming directly to target: rank 1). The blue dotted line indicates chance level (rank 2). Symbols are as in Figure 1.

Accordingly, we only calculated with three possible locations (the ones used in the morning, noon and evening) in determining the rank order of visits to the possible target zones. Theoretically, if rats randomly select among the three possible target zones, this value is 2 on average, while if they perfectly know the actual location it is 1 (i.e. they directly swim to the platform). Results showed, however, that the animals were not able to find out the contextual rule (coupling to the time of the day) of the platform location (Figure 5). LE rats' performance significantly deviated from random choice (mean rank was 2.2 ± 0.04; *χ*_(df = 2)_^2^ = 29.23, *p* < 0.001), but that of LH rats did not (mean rank: 2.0 ± 0.05; *χ*_(df = 2)_^2^ = 0.15, ns).

That is, LH rats found the actual target roughly by the 2^nd^ choice, while LE rats deviated towards a higher value. The reason of this deviation is that LE rats (but not LH rats) tended to visit first the platform location of the previous session (the proportion of first visits to the previous platform location was 47.9% for LE and 37.5% for LH; the former is significantly higher than chance (33.3%), (*χ*_(df = 1)_^2^ = 19.04, *p* < 0.001), while the latter is not (*χ*_(df = 1)_^2^ = 1.09, ns).

### 3.3. 2-Platform Experiment

**Figure 6:**
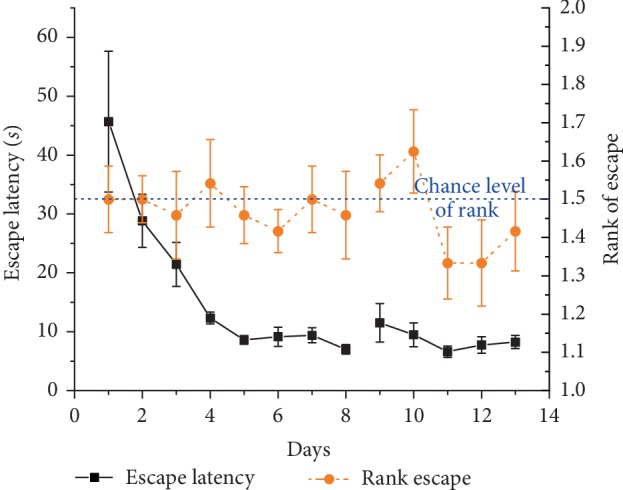
Daily mean ± SEM escape latency (black solid line, left Y-axis) and mean rank ± SEM of visits to the actual target zone out of the two possible locations (orange (dark gray) dashed line, right Y-axis) in trial 1 across 8 + 5 days of 2 daily trials (noon and evening) in the 2-platform experiment of 12 Lister Hooded rats. Measurements on days 9–13 happened after one month break. Rank 1 represents the choice of swimming directly to target, rank 1.5 indicates chance level (shown as blue dotted line).

Escape latency gradually and significantly decreased through the experiment (*F*_(12, 132)_ = 8.5, *p* < 0.001) (Figure 6).

**Figure 7:**
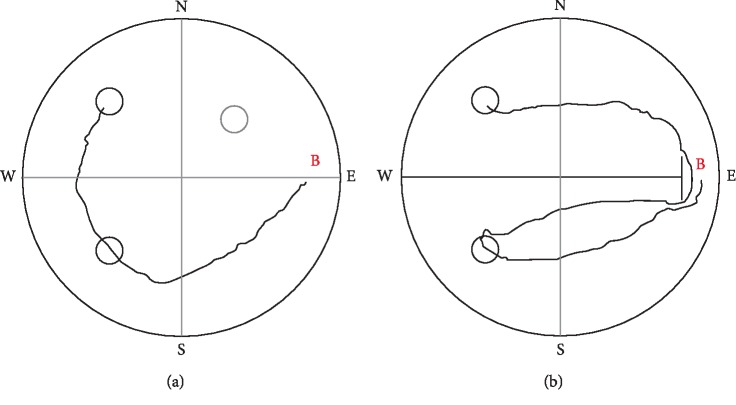
Sample tracks to the target position (NW) from the “East” beginning point (B) of LH rats by sequentially visiting the two potential target zones, (a) in the 2-platform experiment on day 12 (escape latency: about 6 s), (b) in the 2-platform experiment with separation board on day 13 (escape latency: about 14 s).

Again, after a few days rats adapted to the two possible platform sites and ignored the previous morning location (data not shown). They were able to find the platform by sequentially visiting both possible target zones (Figure 7(a)). As there were only 2 possible platform locations in this experiment, the rank of visit to the current target could be 1 or 2. There was no significant difference from chance level (1.5) in this setup (mean rank: 1.5 ± 0.03; *χ*_(df = 1)_^2^ = 0.65, ns) (Figure 6).

### 3.4. 2-Platform + Board Experiment

**Figure 8:**
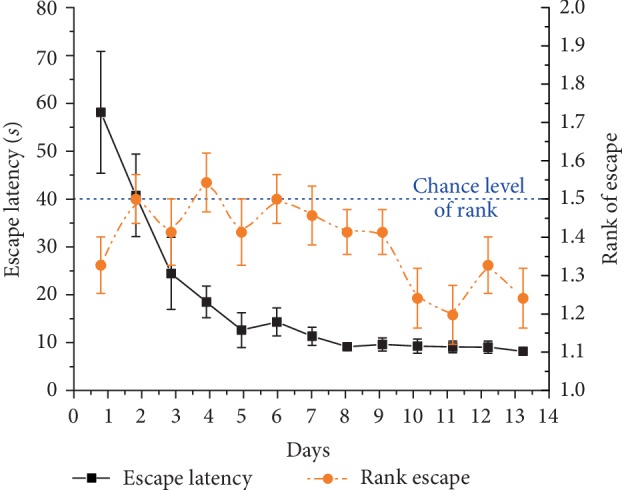
Escape latency (mean ± SEM) of the 1^st^ morning and 1^st^ evening trials across 13 training days in the 2-platform + board experiment of 12 Lister Hooded rats (black solid curve, left Y-axis). Orange (dark gray) dashed line shows the mean rank ± SEM of visits to the actual target zone out of the two possible locations (right Y-axis). Rank 1 represents the choice of swimming directly to target, rank 1.5 (blue dashed line) indicates chance level.

Mean escape latency showed a significant decrease across 13 days (*F*_(12, 132)_ = 8.8, *p* < 0.001) in 2-platform+board experiment in trial 1 (Figure 8). In case the animals did not swim directly to the target, the distance hence the escape latency increased compared to the previous experiment without separation board (Figure 7). The escape latency on day 13 was significantly higher, than that in the 2-platform experiment on day 13 (10.0 ± 0.46 and 7.43 ± 0.95 s), respectively; dependent samples *t*-test: *t*(11) = −2.59, *p* < 0.05).

Animals' performance was significantly better than chance level across the 13 training days (mean rank: 1.39 ± 0.03; *χ*_(df = 1)_^2^ = 7.55, *p* < 0.01). The mean rank did not differ significantly (*χ*_(*df* = 1)_^2^ = 1.13, ns) on the first 9 days (1.45 ± 0.03), but rats showed a better performance on the last 4 training days (mean rank: 1.26 ± 0.05; *χ*_(df = 1)_^2^ = 11.69, *p* < 0.001) (Figure 7).

**Figure 9:**
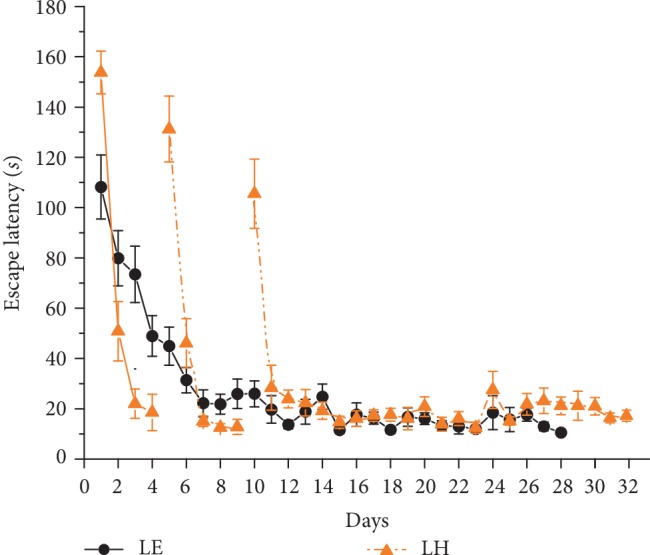
Mean escape latency values ± SEM of the 1^st^ daily trials in the 2-platform + board experiment of 24 Lister Hooded and 35 Long Evans rats with alternating morning/evening sessions. LH rats had 9 pre-training days and 23 training days, whereas LE rats were tested for 28 training days. Symbols are as in Figure 1.

Another cohort of 24 naïve Lister Hooded rats that had not been earlier introduced to the 2-platform- and 3-platform experiments was also tested in the 2-platform + board paradigm, but according to a modified training procedure (see Methods). Switching to another target location at another time of the day on training days 5 and 10 caused a transient increase in escape latency. The improvement in latency measures during 32 days proved to be significant (*F*_(31, 713)_ = 33.2, *p* < 0.001) (Figure 9).

In this task, LE rats, which were tested without pre-training periods, also showed a significant decrease in escape latency (*F*_(27, 918)_ = 19.1, *p* < 0.001) swimming alternately morning and evening sessions from the first test day on (Figure 9). Repeated measures ANOVA revealed significant “days” effect on latencies.

**Figure 10:**
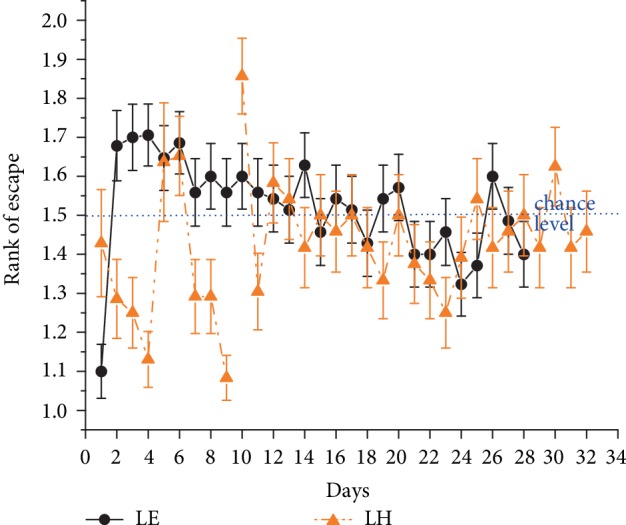
Mean rank of visits ± SEM to the actual target zone out of the two possible locations in trial 1 in the 2-platform+board experiment across 9 pretraining days and 23 training days (LH) or across 28 training days (LE). Rank 1 = swimming directly to target, rank 1.5 (blue dashed line) indicates chance level. Symbols are as in Figure 1.

Considering the test days of alternating morning and evening sessions, the rank of escape values were summarized from the first training day for 24 LH rats and 35 LE rats (Figure 10). None of the strains” performance showed a significant difference from chance: LH: mean rank: 1.4 ± 0.02; *χ*_(df = 1)_^2^ = 2.32, ns; LE: mean rank: 1.5 ± 0.02; *χ*_(df = 1)_^2^ = 1.17, ns.

In the 2-platfrom + board experiment, we also looked at the within session learning of the animals. Particularly, we determined the percentage of animals that (a) used a win-stay strategy, i.e. visited the target location as a first choice both in the “query” trial (trial 1) and in the “confirmatory” trial (trial 2) out of the animals choosing correctly in trial 1; and (b) followed a lose-shift strategy, that is, first chose the incorrect target location in trial 1, but performed properly in trial 2, related to the number of rats swimming to the incorrect location in trial 1. We then averaged these percentage values across the 32 training days of LH and 28 training days of LE rats (Table 1).

### 3.5. 2-Platform + Closed Board Experiment

**Figure 11:**
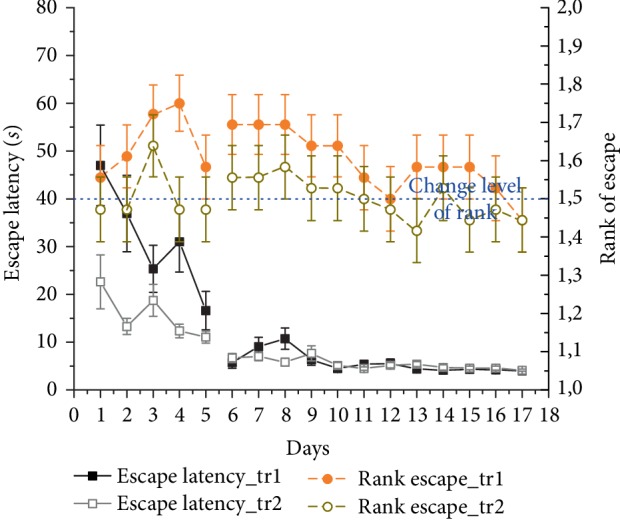
Escape latency (mean ± SEM) of the 1st trials (both in the morning and in the evening) in the 2-platform+closed board experiment of 18 Long–Evans rats (black solid curve, filled symbols, left *Y*-axis), and of the 2^nd^ trials (morning, evening) (gray solid curve, unfilled symbols, left *Y*-axis). On training days 6–17 only escape latencies of rats that found the platform were considered. Orange dashed line and filled circle symbols show the mean rank ± SEM of visits to the actual target zone out of the two possible locations in trial 1 and green dashed line with unfilled circle symbols represent rank ± SEM in trial 2 (right *Y*-axis). Rank 1 represents the choice of swimming directly to target, rank 2 represents the choice of swimming to the wrong direction; rank 1.5 (blue dashed line) indicates chance level.

Mean escape latency showed a significant decrease across the first 5 days, when the corridor was not closed in case of an incorrect choice (repeated measures ANOVA “days” effect (*F*_(4, 64)_ = 4.84, *p* < 0.01), “trials” effect on latencies (*F*_(1, 16)_ = 32.6, *p* < 0.001), interaction: ns) (Figure 11). Animals that chose the correct target position got a rank value of 1, while rats swimming to the incorrect direction or first choosing the correct direction but returning to the incorrect half of the maze before finding the platform, got rank 2. Animals' performance was significantly worse than chance level in trial 1 across the 5 training days (mean rank: 1.64 ± 0.04; *χ*_(df = 1)_^2^ = 7.67, *p* < 0.01).

Punishing the rats after choosing the wrong direction did not improve the rats's performance on days 6–17 (mean rank: 1.60 ± 0.02; *χ*_(df = 1)_^2^ = 9.48, *p* < 0.01). Even in trial 2 the mean rank did not differ significantly from chance level (across the first 5 training days: 1.51 ± 0.04; *χ*_(df = 1)_^2^ = 0.01, ns; across days 6–17: 1.50 ± 0.02; *χ*_(df = 1)_^2^ = 0.00, ns) (Figure 11). Within session learning strategies (win-stay, lose-shift) are separately shown for days 1–5, when the corridor was still open for correction, and for days 6–17, when corridor was closed (Table 1).

The “worse than chance” performance of the animals in the first trials prompted us to examine the intersession changes, i.e. from trial 2 of the previous session to trial 1 of the following session. This analysis revealed that during days 1–5 (90.7 ± 3.4%) of the cases of a correct choice in trial 2 was followed by the choice of the same (but by then incorrect) side in trial 1 of the following session whereas in 38.6 ± 6.0% of the cases of an initial incorrect choice in trial 2 animals swam to the other (by then also incorrect) side in trial 1 of the following session. These values basically did not change during days 6–17 when the incorrect choices were consequently punished (87.5 ± 2.6% and 33.8 ± 4.3%, respectively).

## 4. Discussion

All the tested animals of both strains could quickly learn the standard Morris water-maze task with the platform being in the south-east quadrant. After acquiring this basic knowledge, we modified the protocol to translate the human “orientation” test to rats and query them whether they can determine which time of the day they are. For this purpose, the platform was placed onto different locations depending on the time of the day: morning (NE), noon (NW) or evening (SW). This experiment was carried out one weekend (LH) or one week (LE) after the standard Morris water-maze experiment and despite the displaced platform, on the first day rats could escape in about half of the time than on the first day of the standard Morris experiment. The shorter escape latencies reflect that the animals had already learnt the basic contextual framework of the task, i.e “it is a place where a non-visible underwater platform should be searched for to escape from the water”. Furthermore, during the subsequent sessions they gradually ignored the fourth, formerly used south-east platform position and only searched for the platform in the three “active” locations. That is, LE and LH rats also learnt a second “rule of the game”, namely the only possible locations of the escape platform. In other words, they could connect the “what” and “where” components of the task. However, animals of neither strain were able to find out the third contextual rule of the paradigm: the connection between the time of the day (“when”) and the actual platform location. They used the strategy of sequentially visiting the three potential target zones. Analysis of the rank order of the visits to the possible target locations revealed that LH rats were choosing randomly between them irrespective of the time of the day while the significant inclination of LE rats to first visiting the platform location of the previous session suggests a kind of win-stay strategy instead of choosing completely randomly.

Water-maze based time-of-day (morning and afternoon) discrimination studies found in the literature yielded similar results. In the study of Lukoyanov et al. ad libitum fed Wistar rats failed to acquire the temporal discrimination component of the task but they could seemingly learn where to search for the two possible platform locations [20]. Thorpe et al. reported that LE rats could not learn the time-place connection and were choosing randomly between the correct and the opposite platform location but “spend significantly more time in the combined correct and opposite quadrants compared to combined time spent in the other two quadrants”, that is they “learned the quadrants in which the platforms were located, but not the times in which they were in that quadrant” [21]. Widman et al. found that SPRD rats had not formed a time-place discrimination after 25 days training; they were selecting locations at random [22].

As the rats in our study did not manage to solve the task with three platform locations on three different times of the day, we radically simplified the task. We reduced the number of sessions to two with a one hour longer time interval between them. Furthermore, rats started the trials always from the same starting position and the same distance needed to be travelled to either platform location. Therefore, the platform could be found either by allocentric navigation (relying on extra-maze cues) or by egocentric navigation (following the same swimming pattern). Despite these eased conditions the 12 LH rats tested in this setup still did not manage to discover the time-related rule; they continued to follow a random choice strategy. Correcting an initial wrong choice just required about 2 seconds additional swimming, which, on one hand, suggests the subjects exactly knew the positions of the two possible targets, on the other hand also suggests that this minimized plus effort did not mean a cost high enough to switch to a more effective, albeit mentally more demanding strategy. Even obtaining pellets as a reward in case of correct choice apparently did not sufficiently motivate the animals to follow the rule.

To augment the cost of correcting an initial wrong choice (longer swimming time and distance) we inserted a separation board between the two halves of the maze. Further, we extended the interval between the morning and evening sessions to 8 hours. We assumed if lack of discrimination might be (at least partly) due to a time resolution problem, i.e difficulty in distinguishing the times of the day with 5 or 6 hours interval between them, then the prolonged interval would help the rats to distinguish morning sessions from evening sessions. Unfortunately, none of these modifications brought substantial changes in the performance of the animals. As the above cited water-maze time-place learning studies were carried out with a relatively low number of animals (*n* = 5–8) we extended this experiment for 35 LE and 12 + 24 LH rats with essentially the same outcomes.

A faint signal for an eventual time-place learning was obtained on the last 4 days of the 2-platform + board experiment when the escape rank of 12 LH rats was approaching 1, but this tendency was not replicated with a larger number of animals. However, the latter were trained across 64 trials whereas the “pioneering” LH rats also took part in the 3-platform experiment (32 trials) and 2-platform experiment (52 trials) experiencing altogether 148 time-related “episodes”. It cannot be excluded that they could have shown time-place discrimination with continued training, such as the animals in the study of Lukoyanov et al. which required 200 trials to achieve a performance of 80% correct choices [20].

Lukoyanov et al. found that – in contrast to ad libitum fed rats – food-restricted rats (60% of ad libitum consumption) “were apparently able to learn the time-of-day discrimination” [20]. The authors argued that animals may have relied on a circadian oscillator entrained by the regular daily feeding regime. Our animals were also food-restricted (though to a lesser degree than in the Lukayanov et al. study [20], and regularly had their meal at the end of their active (dark) period. In the 2-platform+board experiment the intersession interval was also similar to that applied by Lukoyanov et al. [20]. Morning sessions followed “wake-up” (lights off) time by 2 hours while evening sessions preceded feeding time by an hour. Thus the rats could have used the temporal information provided by the hypothesized food-entrained or light-entrained circadian system. Apparently, this was not the case as they preferred to choose randomly between the potential platform positions in either session.

The strong preference of our rats for random search was also shown by the finding that in cases when an animal's first choice was a correct one in the “query” trial only about 80% of them used a win-stay strategy and made the same decision for target finding in the “confirmatory” trial commencing only 30 min later. Further, out of rats swimming first to the false target position in trial 1 there were only 53% (LE) or 60% (LH) which used a lose-shift strategy and corrected their initial wrong choice in the second trial. The above percentages were stable during the whole test period. Because of this, the percent correct choices of the rats fell off from 100% in trial 2, reaching only 67.5% (LE) or 70.5% (LH). Thus, “guessing” as a search “strategy” was deeply imprinted in the rats not only between sessions but also—though to a lesser extent—within a session. Inspecting the individual performance of the rats further supported this conclusion: there was no rat in the groups which kept the right swimming direction from trial 1 to trial 2 in each session and also none of them consistently changed its incorrect choice between the two trials.

Widman et al. demonstrated in a climbing tower task that when response cost was sufficiently high rat became more prone to choose time-place discrimination strategy [23]. In the water-maze task, Widman et al. increased response cost by strapping a weight belt on the animals during swimming which resulted in a better than chance discrimination between morning and afternoon sessions after 25 trials [22]. Although, in our experiment the cost of correcting an initial wrong choice more than doubled (in terms of swimming distance and time) compared to the “without board” condition it was apparently not high enough to force the rats to switch from random search to time-of-day discrimination.

Therefore, we further increased the cost of an incorrect choice in the “2 platform + closed board” experiment. Rats that chose an incorrect direction were punished by closing the corridor between the two halves of the maze thus eliminating the possibility of an immediate correction. Animals were confined to the platform-free half for 5 min in trial 1 and 3 min in trial 2. Just as in case of LE and LH animals LEJ2 rats were also able to learn quickly that there were two possible target locations to escape, as mean escape latency dropped to 16 seconds in the initial “run-in” phase of the experiment when they could still correct their wrong choice, and stayed at about 5 seconds on days 6–17 for the animals that swam to the correct target position. Some of the rats that made a false choice even tried to “correct” their decision by vigorous attempts to break through the closed corridor under the water. Nevertheless, even punishing the rats' incorrect choice did not make them master the task.

In this cohort, within session performance of the rats (from trial 1 to trial 2) also showed a dominant win-stay strategy (around 90%), independently of applying punishment or not. However, in contrast to the “2-platform + board” experiment, only about 30% of the rats followed a lose-shift strategy, i.e. corrected their initial false choice at days 1–5, which performance became even worse (~23%) during the punished period. A more striking difference was the significantly worse rank of 1.6 from chance level (rank = 1.5) in the first trials suggesting that the rats preferred to choose the false direction. This kind of performance can be explained by a win-stay strategy from one session to the other. Indeed, after a correct choice in trial 2, 90% of the rats swam to the same target position in trial 1 of the next session, and after an incorrect choice in trial 2, 40% swam to the other, by then incorrect side in trial 1 of the following session (lose-shift).

The reliance of LE rats on random search in the 2-platform+board experiment contrasts our findings in the 3-platform experiment where they showed signs of a win-stay strategy. An explanation for abandoning the latter in the two-choice paradigm may be that the fewer the choice possibilities the higher the success rate of a random choice strategy and the less the cost of an incorrect choice. Another possibility may be that LE rats were 18 months old at the beginning of the 2-platform + board experiment thus might be constrained to mentally less demanding learning strategies. Mulder et al. showed that 17-month-old mice—in contrast to 4-month-old counterparts—were unable to learn de novo a time-place discrimination task [15]. However, as 20-month-old LEJ2 rats did show a win-stay strategy in a very similar two choice assay, a more probable explanation may be that the initial strategy of Long-Evans rats in a choice paradigm may be the win-stay one, but in case of its ineffectiveness they change to a simpler one, namely random choice. In accordance with this assumption, a tendency can be observed toward a chance level performance by time in the first trials of the “2-platform + board” experiment.

Obviously, our rats were not able to acquire time of day discrimination though we assumed that combining three “successful” driving factors from the literature: food restriction, high response cost and extended inter-trial intervals [20, 22, 23], and testing a well-trained rat population with “widespread knowledge” [16] would result in good performance. However, despite their failure to differentiate between times of day animals were able to learn the possible correct locations. This finding is in accordance not only with studies in the water-maze [20, 21], but also with results obtained in food-rewarded time-place learning tasks. In a 4-arm radial maze paradigm, Sprague-Dawley rats did not show time-place learning, but avoided the non-rewarded arms [23]. Carr and Wilkie observed that rats learned to use the active levers and to ignore the inactive ones in a 4-lever Skinner box across a relatively low number of trials. They required further trials to achieve a certain level of time-of-day discrimination where they still produced 25–30% response on the alternate lever but less than 5% on the inactive levers [24]. Means et al. (2000) showed in a *T*-maze task that rats easily acquired a time-of-day related “go – no go” discrimination (they could learn at which time of the day the arms of the T-maze are baited (“active”) and at which time are not (“inactive”) but had difficulties in acquiring a time-of-day choice discrimination, that is, to distinguish which arm is baited at a given period. They concluded that rats rather use time of day as an occasion-setting stimulus but not as a signal for a specific response [25].

The generally observed poor time-place learning performance of rats could source from an evolutionary built in, conservative mechanism. However, it is a question whether it characterizes the rat as an opportunistic species or this ability may have only lost in the laboratory rats due to an environment with complete lack of evolutionary pressure to distinguish different times of day. This assumption could be tested by investigating wild-caught rats within laboratory conditions.

In summary, we could not translate the “orientation” task of human cognitive inventories to a time-place discrimination paradigm in the laboratory rat. It might have been possible by using a much longer training procedure; however, in the framework of our cognitive modelling where the animals take part parallel in many different cognitive tasks across their lifespan we would have needed a relatively short-term method resistant to interference with other learning tasks. A possible alternative solution could be the utilization of the newly developed housing technologies where various learning tasks, including those requiring episodic-like memory, can be implemented and studied in the home-cages of the animals.

## Figures and Tables

**Table 1:** Intertrial changes in target finding of LH, LE and “LEJ2” rats depending on the possibility of correcting the initial false choice. Win-stay strategy: percentage ± SEM of rats swimming to the correct location in trial 2 as in trial 1 related to the number of rats choosing correctly in trial 1. Lose-shift strategy: percentage ± SEM of rats swimming to the correct location in trial 2 after choosing the incorrect one in trial 1 related to the number of rats swimming to the incorrect location in trial 1.

Possibility to correct	Cohort	Win-stay (%)	Lose-shift (%)
Yes	LE	82.4 ± 1.9	52.7 ± 2.4
	LH	80.7 ± 2.4	60.3 ± 3.0

Yes (days 1–5)	LEJ2	87.3 ± 4.2	27.9 ± 6.2
No (days 6–17)	LEJ2	92.0 ± 2.0	22.9 ± 2.7

## Data Availability

The data used to support the findings of this study are available from the corresponding author upon request.
